# Coronary artery calcium on lung cancer radiation planning CT aids cardiovascular risk assessment

**DOI:** 10.1186/s40959-024-00283-5

**Published:** 2024-11-12

**Authors:** Matthew Lui, Noah Kim, Raja Zaghlol, Pouya Joolharzadeh, Elena Deych, Clifford Robinson, Shahed Badiyan, Pamela K. Woodard, Joshua D. Mitchell

**Affiliations:** 1 General Medical Sciences, Washington University School of Medicine, St. Louis, MO USA; 2 Cardiovascular Division, Department of Internal Medicine, Washington University School of Medicine, St. Louis, MO USA; 3 Stony Brook School of Medicine, Stony Brook University, Stony Brook, NY USA; 4 Department of Radiation Oncology, Siteman Cancer Center, Washington University School of Medicine, Barnes Jewish Hospital, St. Louis, MO USA; 5 Mallinckrodt Institute of Radiology, Washington University School of Medicine, Barnes-Jewish Hospital, St. Louis, MO USA; 6 Cardio-Oncology Center of Excellence, Cardiovascular Division, Washington University School of Medicine, St. Louis, MO USA; 7 Division of Cardiology, Washington University in St. Louis, 660 S. Euclid Ave, CB 8086, St. Louis, MO 63110 USA; 8 Department of Radiation Oncology, UT Southwestern Medical Center, Dallas, TX, United States

**Keywords:** Coronary artery calcification, Non-small cell lung cancer, Radiation therapy

## Abstract

**Background:**

Patients with non-small cell lung cancer (NSCLC) undergoing thoracic radiation are at high cardiovascular risk. Semiquantitative assessment of coronary artery calcification (CAC) on baseline planning non-gated chest computed tomography (CT) scans may help further risk stratify patients.

**Objectives:**

This study aimed to characterize the association between CAC and major adverse cardiovascular events (MACE; myocardial infarction or stroke) and assess the utility of semiquantitative assessment of CAC.

**Methods:**

Patients with NSCLC with non-contrast planning chest CT scans were evaluated for CAC. Planning scans were visually graded using the CAC-DRS method, stratifying patients into no, mild, moderate, and severe CAC groups. Demographics, comorbidities, and radiation treatment characteristics were gathered, and CAC groups were assessed for the incidence of MACE after initiation of radiation therapy.

**Results:**

Out of 137 patients, 39 patients had no CAC, and 98 patients had any CAC (38 with mild CAC, 34 with moderate CAC, and 26 with severe CAC). There was 1 MACE event in the no CAC group and 11 in patients with any CAC. The presence of CAC was associated with increased MACE compared to no CAC (*p* = 0.034). Semiquantitative CAC analysis correlated with formal CAC scoring.

**Conclusion:**

There is a significantly lower incidence of MACE in patients with no CAC on planning CT compared to patients with higher burdens of CAC. CAC burden is an important risk factor for adverse cardiovascular events in patients with NSCLC undergoing thoracic radiation. Semiquantitative CAC scoring may be a useful proxy when formal CAC scoring is unavailable.

## Background

While a vital component of treatment for many patients with non-small cell lung cancer (NSCLC), thoracic irradiation is associated with an increased risk of coronary artery disease (CAD) and coronary-related death [1–5]. Patients with NSCLC receive large doses of thoracic irradiation and are thus at especially high risk for cardiac toxicity. In a series of 322 patients with locally advanced NSCLC undergoing irradiation at Washington University, 59.2% of patients had cardiac toxicity of grade 1 or higher [3]. Within the cohort, the cardiac dose delivered – a function of tumor size, tumor location, and other patient factors – was associated with both cardiac toxicity and overall survival [3]. Adding to the risk of the radiation, patients with lung cancer are more likely to have underlying cardiac comorbid risk factors such as smoking at time of initial diagnosis [5]. Unfortunately, these patients are often undertreated for their cardiovascular disease. In one study of 168 patients with NSCLC, 62% of the 78 patients with coronary artery calcifications (CAC) on their baseline computed tomography (CT) scan went untreated with statin therapy [6].

Patients undergoing radiation therapy all have a baseline, planning computed tomography that provides a unique opportunity to assess for coronary artery calcium, improve a patient’s cardiovascular risk stratification, and target preventive therapy. The presence of CAC on CT is proportional to a patient’s atherosclerotic burden and is associated with higher incidence of adverse cardiovascular events in the general population [7, 8] and in patients with cancer [9, 10]. In patients with breast cancer, CAC on baseline CT prior to radiation was significantly associated with 9-year cumulative incidence of acute coronary events post-radiation [9]. Additionally, in breast cancer patients with no known CAD, CAC on surveillance CT, but not Framingham risk score, predicted the composite endpoint of all-cause mortality and cardiac events [10]. Early evidence suggests that CAC on baseline CT prior to lung cancer radiation also identifies patients at highest risk for cardiotoxicity in patients with NSCLC [11].

While CAC is historically assessed using the formalized Agatston method on dedicated ECG-triggered CT scans [12], CAC seen on non-gated, non-contrast CT scans correlates with formal gated CAC scans and has significant predictive value [13, 14]. In a meta-analysis of 3 studies with 661 participants, the agreement in calcium score between non-triggered and ECG-triggered CT was 0.94 (95% CI, 0.89–0.97). Additionally, there is evidence that semiquantitative assessment of CAC using the visual CAC-DRS method may be adequate to characterize cardiovascular risk [15]. However, validation of this approach in in a cohort of patients undergoing radiation therapy is needed.

We sought to characterize the ability of pre-existing CAC on non-electrocardiogram (ECG)-gated planning CT to predict future adverse cardiac events in a registry of patients with non-small cell lung cancer with extensive radiation treatment data. In order to assess the utility of a physician’s subjective interpretation of CAC on a planning CT, we also aimed to characterize the association between a visual, semiquantitative scoring method vs. a computed, quantitative scoring method in assessment of overall CAC burden.

## Methods

### Study population

A single institution retrospective review was conducted on 509 patients with non-small cell lung cancer treated with definitive radiation therapy with or without chemotherapy at a tertiary care center between January 2001 to December 2014. This review was conducted on an Institutional Review Board approved, retrospective registry of locally advanced lung cancer [3]. Patients were excluded if they did not have an available non-contrast planning chest CT for review or if there was no accessible documentation regarding follow up after their planning chest CT. Planning chest CTs were performed using Philips Brilliance Big Bore CT scanners (Philips, Amsterdam, Netherlands) with slice thickness of 3 mm and radiation dose ranging from 13.2 to 40.1 mGy. Of the initial cohort, 295 patients had planning chest CT scans in our system available for review. 173 of these patients had non-contrasted chest CT studies. Of these patients, 137 patients had follow-up data in the electronic medical record. These 137 patients comprised the cohort used for analysis. This study was approved by the local Institutional Review Board, and informed consent was not required due to the retrospective nature of the study.

Baseline comorbidities and demographic information were extracted using International Classification of Disease-Ninth Revision (ICD-9) and International Classification of Disease-Tenth Revision (ICD-10) codes from the institutional electronic health record (Epic Systems, Verona, WI) for any inpatient or outpatient diagnoses entered before the date of planning chest CT. These characteristics were confirmed through manual chart review.

### Coronary artery calcium scoring

CT images were reviewed for the presence and severity of coronary artery calcification using a visual, semiquantitative scoring system as well as by processing the DICOM images through the Vital software system (Canon Medical, Minnetonka). The visual, semiquantitative system was designed to mimic what can be done by a clinician’s office in the absence of requiring post-processing protocols. Visual scoring was performed using the CAC-DRS method, stratifying patients into no, mild, moderate, and severe CAC groups. The Vital software program was used to analyze DICOM images to provide formalized Agatston scoring, and scores were categorized according to the CAC-DRS cutoffs (no CAC: 0, mild CAC: 1–99, moderate CAC: 100–299, severe CAC: >300). Computed calcium scores were compared to visual scores by three scorers to assess reliability between the two methods.

### Outcome measures

The primary outcome measured was time to the first major adverse cardiovascular event (MACE) that occurred after initiation of radiation therapy adjusting for the competing risk of non-cardiac death. MACE was defined as myocardial infarction, coronary revascularization, and stroke. The primary analysis compared the incidence of MACE in patients with no CAC compared to patients with any CAC. Secondary analyses compared patients across CAC groups. Patients were followed until the occurrence of the outcome, disenrollment from the health care system, or the end of the study on January 1, 2021. All charts were manually reviewed for MACE events through the study end as defined by the treating providers.

### Statistical analysis

Clinical and demographic information were described using standard descriptive statistics. Categorical variables were compared using the chi-square test and continuous variables were compared using t-test or Wilcoxon test, as appropriate. Cause-specific analysis with Cox proportional hazard models assessed the effect of CAC compared to no CAC on the primary outcome of MACE. Additional analysis evaluated the effect of CAC severity on outcome in four groups (no CAC and mild, moderate, and severe CAC). Potential covariates were evaluated univariately for inclusion in Cox proportional hazard models to determine the association of CAC burden with the outcomes. Age and sex were forced into the multivariable model due to their known association with CAC and severity. Proportionality assumptions for Cox model were tested by Schoenfeld residuals. Inter-observer reliability between visual scores and computed scores was assessed through the kappa statistic and Kendall correlation.

## Results

Of the 137 patients included for analysis, 74 (53.6%) were male (mean age 65.2 +/- 9.7). The baseline demographics and comorbidities are further described in Table 1.

**Table 1:** Patient demographics

	Overall (*n* = 137)	No CAC (*n* = 39)	Any CAC (*n* = 98)	*p*-value
Male Sex (%)	74 (53.6)	15 (38.5)	58 (59.2)	0.045
Age at Radiation (mean (SD))	65.22 (9.72)	57.14 (8.33)	68.55 (8.23)	< 0.001
Race (%)				0.445
Black	32 (23.2)	12 (30.8)	20 (20.4)	
Other/Unknown	12 (8.7)	3 (7.7)	9 (9.2)	
White	94 (68.1)	24 (61.5)	69 (70.4)	
Hypertension (%)	73 (52.9)	12 (30.8)	60 (61.2)	0.002
Hyperlipidemia (%)	34 (24.6)	3 (7.7)	31 (31.6)	0.007
Coronary Artery Disease (%)	31 (22.5)	0 (0.0)	31 (31.6)	< 0.001
Type II Diabetes Mellitus (%)	34 (25.0)	6 (15.4)	28 (29.2)	0.146
Congestive Heart Failure (%)	12 (8.8)	1 (2.6)	11 (11.5)	0.179
Cancer Histology (%)				0.319
Adenocarcinoma	69 (50.0)	18 (46.2)	50 (51.0)	
NSC	20 (14.5)	9 (23.1)	11 (11.2)	
Other	1 (0.7)	0 (0.0)	1 (1.0)	
Squamous Cell Carcinoma	48 (34.8)	12 (30.8)	36 (36.7)	
Smoker (%)				0.27
Current	53 (38.4)	16 (41.0)	36 (36.7)	
Former	77 (55.8)	19 (48.7)	58 (59.2)	
Never	8 (5.8)	4 (10.3)	4 (4.1)	
PAD (%)	6 (4.3)	2 (5.1)	4 (4.1)	1
Cancer Stage (%)				0.216
Recurrent	5 (3.6)	1 (2.6)	4 (4.1)	
Stage II-a	3 (2.2)	0 (0.0)	3 (3.1)	
Stage II-b	4 (2.9)	1 (2.6)	3 (3.1)	
Stage III-a	85 (61.6)	20 (51.3)	64 (65.3)	
Charlson Comorbidity Score (mean (SD))	3.51 (1.95)	2.11 (1.45)	4.07 (1.84)	< 0.001
Heart dose radiation, mean (median [IQR])	19.32 [12.09, 28.84]	21.34 [10.91, 30.42]	19.27 [12.09, 27.73]	0.757
Heart dose radiation, max (median [IQR])	70.03 [64.55, 75.10]	66.30 [62.12, 72.50]	70.67 [64.90, 75.85]	0.039
Average Follow up length (years), (median [IQR])	1.33 [0.50, 3.52]	2.23 [0.74, 8.27]	1.17 [0.38, 2.56]	0.009

In visual analysis using the CAC-DRS methodology by observer 1, 39 patients had no CAC and 98 patients had CAC on their planning CT (38 with mild CAC, 34 with moderate CAC and 26 with severe CAC). Over a median follow up of 1.44 (1.05–2.11) years after initiation of radiation therapy, 11 total MACE events were recorded (Table 2), with 1 MACE event in the no CAC group and 10 MACE events in the any CAC group (5 events in the mild CAC group, 2 events in the moderate CAC group, and 3 events in the severe CAC group.) Of note, the single MACE event in the no CAC group occurred 14 years after radiation initiation while 94% of patients had less than 10 years of follow-up. The median heart dose across groups was 21 Gy (Interquartile Range (IQR) 11–30) in the no CAC group and 19 Gy (IQR 12–28) in the any CAC group.

**Table 2:** MACE events

	No CAC (*N* = 39)	Any CAC (*N* = 98)
Myocardial Infarction	1	4
Stroke	0	6
Total MACE Events	1	10

### Time-to-event analysis

In time-to-event analysis, none of the potential confounders (age, sex, HTN, HLD, Diabetes, mean heart dose, max heart dose, smoking) were significantly associated with MACE (Table 3).

**Table 3:** Univariate analysis of potential confounders

	Hazard Ratio	LCL	UCL	*p*-value
Male Sex	0.77	0.21	2.76	0.685
Age at Radiation	1.01	0.95	1.08	0.736
HTN	0.39	0.10	1.51	0.173
HLD	0.34	0.04	2.66	0.302
T2DM	0.35	0.04	2.78	0.319
Heart max dose	1.02	0.96	1.08	0.596
Heart mean dose	1.00	0.94	1.06	0.883
Active Smoker	1.19	0.33	4.25	0.785

**Fig. 1:**
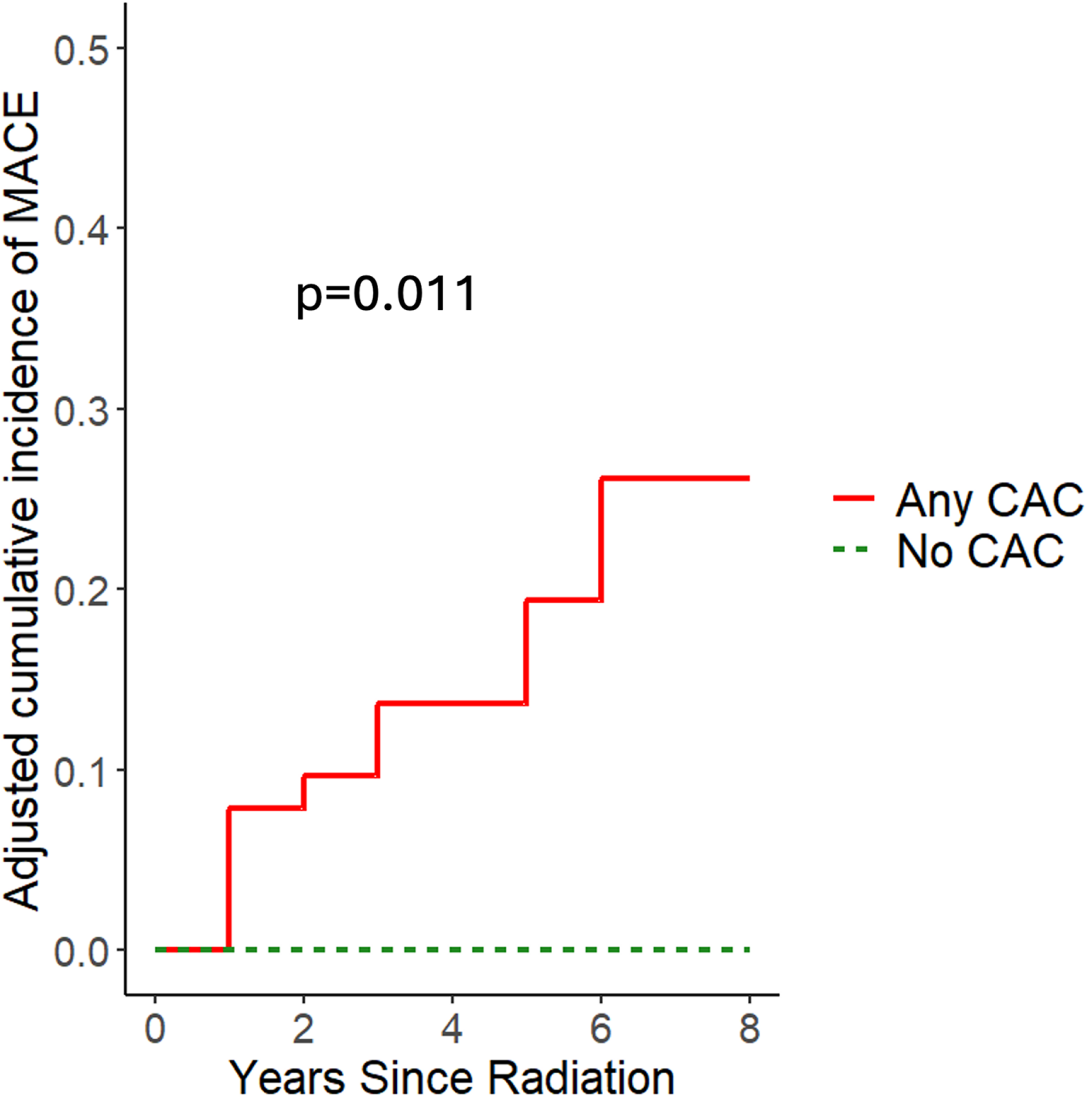
Cumulative incidence of MACE in No CAC vs. any CAC adjusted for age and sex

Using a Cox proportional hazards model adjusting for age and sex, the presence of CAC was associated with increased MACE compared to no CAC (*p* = 0.0011; Fig. 1).

**Fig. 2:**
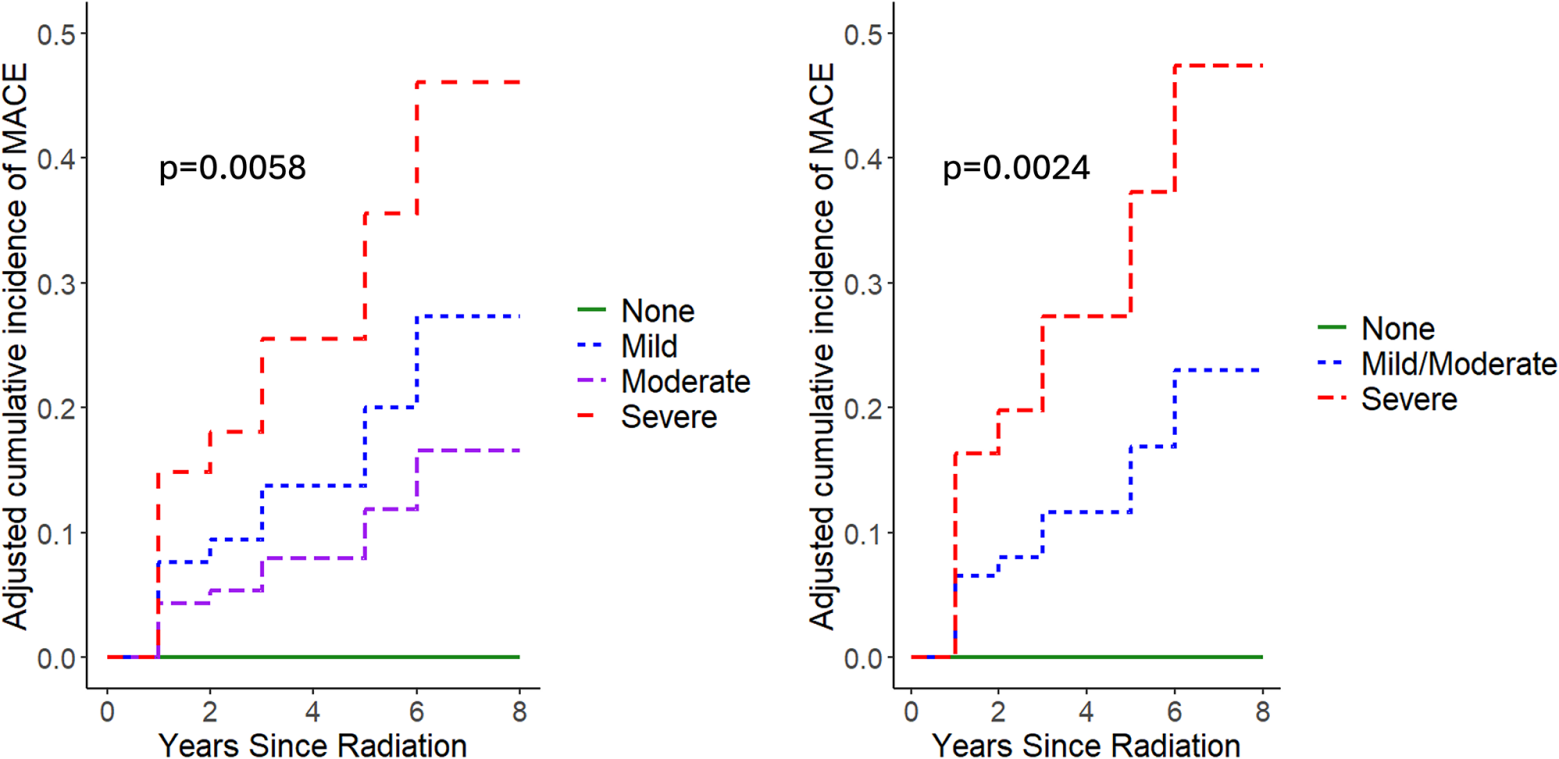
Cumulative incidence of MACE by CAC severity adjusted for age and sex

Evaluating CAC severity and hazard of MACE, there was a difference among groups adjusted for age and sex (*p* = 0.0058, Fig. 2), but there was overlap in the mild and moderate curves. Given the apparent clear separation of severe CAC, a post-hoc analysis was completed combining mild and moderate severity into one group. In this post-hoc analysis, there was a difference among the three CAC groups (no CAC, mild/moderate CAC, and severe CAC) and the hazard of MACE (*p* = 0.0024, Fig. 2), adjusting for age and sex. Severe CAC (adjusted hazard ratio (aHR) 45, 95% CI 2-7509) and mild/moderate CAC (aHR 21, 95% CI 2-2911) had significantly higher hazard of MACE than no CAC, although there was no significant difference between severe and moderate CAC (aHR 2.1, 95% CI 0.33–11.36). Notably, the confidence intervals were wide comparing severe CAC and moderate CAC to no CAC given lack of events in the no CAC group, making it difficult to make a precise estimate for the aHR.

### Inter-observer variability with CAC-DRS

Inter-observer analysis demonstrated good inter-observer reliability using the CAC-DRS methodology for visual CAC scoring. The association of scores between scorer 1 and scorer 2 had a kappa statistic of 0.73 and Kendall correlation of 0.78 (*p* < 0.0001). The association of scores between scorer 1 and scorer 3 had a kappa statistic of 0.62 and Kendall correlation of 0.80 (*p* < 0.0001). The association of scores between scorer 2 and scorer 3 had a kappa statistic of 0.53 and Kendall correlation of 0.77 (*p* < 0.0001). Scorer 1 was used to classify patients into CAC groups for MACE analysis.

### Correlation between CAC-DRS and Agatston score

**Fig. 3:**
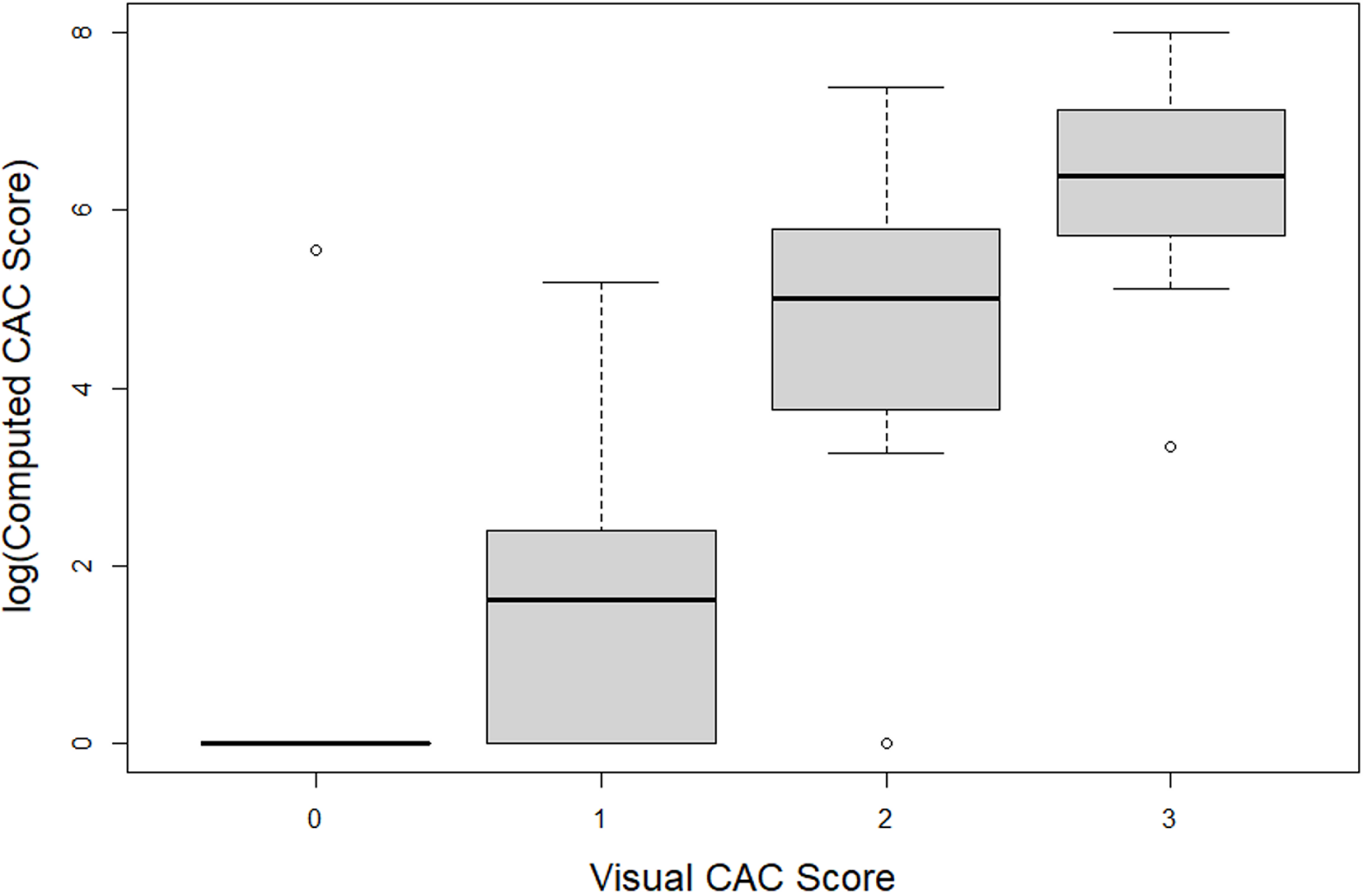
Box plot demonstrating the correlation between visual score and computed score

Seventy-one patients had radiation planning CT scans that were of suitable quality for grading using the VITAL software. The remaining scans were most often unable to be processed by VITAL due to variable slice thickness. The associations between the formal Agatston score computed by VITAL software (using the CAC cutoffs as defined in CAC-DRS) and scorers 1, 2, and 3 were assessed via Kendall correlation and were 0.72. In the 71 patients with scans that could be graded, 6 patients had MACE events (3 patients with stroke and 3 patients with MI). The association between visual scoring and computed scoring is visually depicted in Fig. 3. There were insufficient events to statistically assess the predictive ability of the semi-quantitative CAC scoring (CAC-DRS) compared to the CAC scoring utilizing VITAL.

## Discussion

This study serves as proof-of-concept that CAC can be effectively measured from routinely acquired, non-gated planning chest CT scans through visual assessment in patients with non-small cell lung cancer. CAC burden on the planning chest CT appears to be an important risk factor for adverse cardiovascular events in patients undergoing radiation therapy and was a better predictor than mean radiation dose in our cohort. We observed that the absence of CAC on planning CT was associated with a low baseline incidence of adverse cardiovascular events. The presence of CAC was associated with an increased incidence of MACE when compared to patients without CAC. The semi-quantitative CAC-DRS CAC score also correlated well with computerized CAC scoring in the subset of patients with planning CTs of sufficient quality to be analyzed by the VITAL software.

This study demonstrated that a visual, qualitative assessment of planning chest CTs may be useful to risk stratify patients for adverse cardiovascular events. Visual assessment of CAC burden is a quick, point-of-care risk stratification tool that can be easily performed in the clinician’s office. As such, identifying patients with CAC burden on planning CT during radiation planning offers a window for cardiovascular risk factor modification, including aspirin and statin therapy, counseling on smoking cessation and cardiac rehabilitation. Prior studies have reported that in a similar population, patients with high cardiovascular risk are often incompletely medically optimized with guideline-based anti-lipid therapy [16]. The identification of CAC on lung cancer workup or radiation therapy planning imaging can prompt clinicians to initiate a cardiovascular workup and optimize medical management. In addition, prompt risk stratification may allow physicians to determine which patients may be able to avoid the addition of medications such as aspirin and statins, thus reducing medication burden and polypharmacy. In the general population, a CAC score > 100 identifies patients most likely to benefit from statin therapy [17]. Further research will be needed to identify the threshold for statin benefit in patients who have undergone radiation.

In an analysis of the Multi-Ethnic Study of Atherosclerosis (MESA) [18] cohort, Criqui et al., noted that increased CAC volume, defined by higher Agatston scores, was a positively and independently associated with increased coronary heart disease and cardiovascular disease risks [19]. In our cohort, there was a significant difference in MACE between CAC severity groups, primarily driven by the lack of events in the no CAC group within the analysis window. There was no clear difference and even overlap between the mild and moderate CAC groups. This latter finding may have been impacted by smaller sample size but may also be due to decreased sensitivity to differentiate between mild and moderate CAC on visual assessment. In either case, it would be important to further investigate whether it is more appropriate to classify patients into 3 CAC groups (no CAC, mild/moderate CAC, and severe CAC) in future studies. When mild and moderate are combined, there appears to be increased incidence of MACE with increasing CAC severity which aligns with the MESA cohort [18]. Within the confines of our follow up period (median 3 years), the presence of CAC vs. no CAC appeared to be a stronger predictor of adverse events than CAC severity, though severe CAC was associated with the highest risk of MACE.

The association between coronary calcification on planning CT and risk for future cardiovascular events has been previously described in a similar population of patients undergoing radiation therapy for NSCLC [20, 21]. A retrospective cohort study of 109 patients found that the presence of coronary calcifications on simulation CT scans as quantified using computational software was associated with increased risk of cardiac toxicity, defined as symptomatic pericardial effusion, pericarditis, unstable angina, myocardial infarction, significant arrhythmia, and/or heart failure [20]. This study used MIM software (Cleveland, OH) to calculate coronary artery calcification burden. Another retrospective cohort study of 428 patients with NSCLC demonstrated that an automated scoring model was effective at measuring CAC on planning CT and that higher CAC burdens was associated with mortality. Our findings demonstrate similar associations of CAC with adverse events, while demonstrating that a visual scoring method may be adequate for risk stratification. Visual estimation of CAC burden may be preferable to some clinicians who do not have access to advanced software to perform calcium scoring, while allowing for initial risk stratification and referral for further cardiovascular workup. In addition, less training is needed for visual estimation and may even be utilized by non-cardiologists, such as primary care physicians and radiation oncologists to support preventive measures. Additionally, as evident by our own study, current radiation planning scans are not always able to be analyzed by commercially available software (only 71 of 137 patients had CT scans that were able to be analyzed by VITAL in our study). This approach may be beneficial for radiation oncologists as an accessible method to easily identify patients who could benefit from referral to a cardiologist or cardio-oncologist.

In patients with lung cancer being treated with radiation therapy, the risks of future MACE must certainly be balanced with the risks of inadequately radiating lung cancer. It is increasingly clear that this balance is important given the increased rate of cardiac and all-cause mortality at higher radiation doses. Improved identification of patients at highest risk for cardiovascular events will improve our prognostication and provide opportunities for intervention. In this study, the presence of CAC on planning CTs was more important than the radiation dose delivered to the heart in the confines of the study follow-up period (median 3 years). Neither mean heart dose nor max heart dose were associated with an increase in adverse events during the follow-up period. While radiation dose is known to increase risk for future cardiovascular events, the risk is highest at higher radiation doses and over longer-term follow-up. In addition, coronary artery radiation dose is associated with increased mortality [22] and may serve as a stronger indicator of cardiac risk than overall heart dose. Further studies investigating the interplay of CAC in specific vessels and vessel-specific radiation dosing on adverse cardiovascular outcomes may offer clarity on overall cardiac risk in this patient population. It is not surprising, but an important take-away, that CAC at baseline, a sign of pre-existent cardiovascular disease, is a more important risk factor for future cardiovascular events.

### Limitations

This study was limited to a single center, retrospective evaluation of patients undergoing radiation therapy for NSCLC. As we did not attempt visual CAC scoring on contrast-enhanced CT scans, our overall cohort size was reduced, thus limiting observed MACE events and total power of the study. Excluding contrast-enhanced CT scans may have introduced a selection bias, as clinical differences between patients who underwent contrast-enhanced CT scans and non-contrast CT scans were not characterized. In addition, the limited follow-up period (median 3 years) may have prevented the identification of late cardiovascular events associated with radiation dosing, which is a known risk factor for cardiovascular morbidity and mortality. Further studies controlling for other comorbidities will help clarify the independent risk conferred by high CAC burden and whether it is optimal to combine mild and moderate groups. Large scale studies with multiple reviewers will add reliability to visual assessment methods.

## Conclusion

There is a significant difference in adverse cardiovascular events between patients with no CAC on planning CT and patients with higher burdens of CAC. A simple visual review of CAC burden is an important risk factor for adverse cardiovascular events in patients with NSCLC undergoing thoracic radiation. This simple visual review of CAC burden appears to be an important tool for cardiovascular risk determination in the absence of formal CAC scores.

## Data Availability

The datasets used and/or analysed during the current study are available from the corresponding author on reasonable request.

## Abbreviations

aHR: Adjusted hazard ratio
CAC: Coronary artery calcification
CAD: Coronary artery disease
CT: Computed tomography
ECG: Electrocardiogram
ICD-9: International Classification of Disease-Ninth Revision
ICD-10: International Classification of Disease-Tenth Revision (ICD-10)
IQR: Interquartile Range
MACE: Major adverse cardiovascular events
MESA: Multi-Ethnic Study of Atherosclerosis
NSCLC: Non-small cell lung cancer
